# Natural killer cell subpopulations in the peripheral blood of single ventricle/hypoplastic left heart syndrome patients via single-cell RNA sequencing

**DOI:** 10.3389/ebm.2025.10524

**Published:** 2025-08-14

**Authors:** Hui-Qi Qu, Kushagra Goel, Kayleigh Ostberg, Diana J. Slater, Fengxiang Wang, James Snyder, Cuiping Hou, Garnet Eister, John J. Connolly, Michael March, Joseph T. Glessner, Charlly Kao, Hakon Hakonarson

**Affiliations:** ^1^ The Center for Applied Genomics, Children’s Hospital of Philadelphia, Philadelphia, PA, United States; ^2^ Department of Pediatrics, The Perelman School of Medicine, University of Pennsylvania, Philadelphia, PA, United States; ^3^ Division of Human Genetics, Children’s Hospital of Philadelphia, Philadelphia, PA, United States; ^4^ Division of Pulmonary Medicine, Children’s Hospital of Philadelphia, Philadelphia, PA, United States; ^5^ Faculty of Medicine, University of Iceland, Reykjavik, Iceland

**Keywords:** NK cells, peripheral blood mononuclear cells, single-cell RNA sequencing, single ventricle/hypoplastic left heart syndrome, transcriptome

## Abstract

Natural Killer (NK) cells are integral components of the innate immune system, recognizing and eliminating virus-infected cells. They may play a crucial role in the immune response and contribute to the complications associated with Single Ventricle/Hypoplastic Left Heart Syndrome (SV/HLHS). Utilizing single-cell RNA sequencing (scRNA-seq), NK cells from peripheral blood mononuclear cells (PBMCs) were analyzed in three de-identified SV/HLHS cases and three healthy controls. This study identified two novel NK cell subpopulations that could not be detected by conventional scRNA-seq pipelines or traditional flow cytometry. These subpopulations exhibit distinct gene expression profiles linked to the heterogeneity of immune responsiveness and stress adaptation in NK cells. In SV/HLHS patients, one cluster showed a significant upregulation of androgen response and downregulation of heme metabolism compared to healthy controls. Our study offers new insights into the fine-tuning of immune modulation that could help mitigate complications in SV/HLHS. It suggests that while NK cells in SV/HLHS adapt to support survival in a challenging physiological environment, these adaptations may compromise their ability to effectively respond to additional stresses, such as infections and inflammation.

## Impact statement

This study identifies two previously unrecognized natural killer (NK) cell subpopulations that were undetectable using conventional methods. By applying single-cell RNA sequencing with sophisticated analytical approaches, we provide definitive molecular evidence of their existence and functional relevance. These subpopulations display distinct gene expression patterns, offering new insights into immune system variability and stress adaptation in NK cells. In patients with single ventricle/Hypoplastic Left Heart Syndrome (SV/HLHS), we identify an NK cell subset with increased androgen signaling and decreased heme metabolism, a shift that is significantly more pronounced compared to healthy controls. These findings advance our understanding of immune adaptation in congenital heart disease and suggest that while NK cells compensate for physiological stress, their altered state may reduce their ability to respond to secondary challenges such as infections and inflammation. This work provides a foundation for future research on immune modulation in SV/HLHS and potential therapeutic interventions.

## Introduction

Natural Killer (NK) cells are integral components of the innate immune system, renowned for their ability to identify and eliminate viral-infected cells and tumor cells without prior sensitization [1]. They serve as a first line of defense by exerting cytotoxic effects and producing cytokines that modulate adaptive immune responses [2]. The functionality and population of NK cells are critical for maintaining immune homeostasis and effective immunosurveillance.

Single-ventricle physiology encompasses several rare congenital heart defects and is estimated to occur in approximately 4–8 per 10,000 live births, representing roughly 7.7% of all congenital heart defects [3]. Hypoplastic Left Heart Syndrome (HLHS) is the most common form of functional single-ventricle disease. SV/HLHS results in reliance on the right ventricle to support systemic circulation [4]. Patients with SV/HLHS undergo a series of palliative surgeries to reconstruct the heart’s anatomy and improve hemodynamics [4]. Despite surgical advancements, these patients often experience complications such as increased susceptibility to infections, protein-losing enteropathy, thrombosis, and chronic inflammation [5]. NK cell number and function may be compromised in individuals with SV/HLHS due to factors such as chronic hypoxia, repeated surgical trauma, lymphatic dysfunction, and nutritional deficiencies [6, 7]. These issues may impair NK cell development, reduce cytotoxic capacity, and weaken the immune response, which may explain why SV/HLHS patients are more vulnerable to infections and postoperative complications, and may also contribute to systemic inflammation, worsening heart failure and overall morbidity.

In our study of single-cell RNA sequencing (scRNA-seq) from peripheral blood mononuclear cells (PBMCs) of patients with SV/HLHS, we found that gene expression in NK cells is more closely correlated with SV/HLHS than in other cell types by weighted gene co-expression network analysis (WGCNA). In congenital heart diseases (CHD) including SV/HLHS, NK cells play a crucial role in managing infections and mediating complications related to CHD [8]. In our previous study, we identified 1,600 genes that showed differential expression (DE) in NK cells of SV/HLHS patients [9]. Our current investigation suggests that NK cells in SV/HLHS patients are heterogeneous and potentially receptive of different types of immune modulation. We hypothesized that the SV/HLHS-related DE genes might be affected differently across specific NK cell subpopulations, instead of uniformly across all NK cells. To test this hypothesis, we delved into the single cell transcriptome of NK cells in greater detail.

## Materials and methods

### scRNA-seq of PBMCs

This study received approval from the Institutional Review Board at the Children’s Hospital of Philadelphia (CHOP). PBMCs from three de-identified children (2 males and 1 female) with SV/HLHS were compared to those from three healthy controls (2 males and 1 female). The study design included three biologically independent SV/HLHS cases and three matched healthy controls, providing three replicates per group. This level of replication is commonly accepted in exploratory studies, where the primary goal is to identify robust and biologically meaningful patterns. Blood samples were collected in EDTA-coated tubes and promptly processed at the Center for Applied Genomics (CAG) at CHOP. PBMCs were isolated using Ficoll density gradient centrifugation. scRNA-seq for each sample was conducted using the 10X Chromium Single Cell Gene Expression assay (10x Genomics, Single Cell 3' v3) [10]. Sequencing was performed on the Illumina HiSeq2500 SBS v4 platform. The resulting data from the Chromium scRNA-seq were processed with the Cell Ranger 7.1.0 software suite (10x Genomics), with sequencing reads aligned to the GRCh38 reference genome. The number of cells sequenced and analyzed for each sample ranged from 4,598 to 14,298.

### Data analysis tools

The scRNA-seq data were analyzed using the Seurat R package (v 5.1.0) [11, 12]. Cells were retained if they had more than 200 and fewer than 5,000 detected genes and <15% mitochondrial‐gene reads. Gene expression was normalized with the LogNormalize method (scale factor = 10,000). We identified the top 2,000 variable genes with the variance-stabilizing transformation (vst) method, scaled all genes, and performed principal-component analysis (PCA). The first 10 PCs were used to construct a shared-nearest-neighbor (SNN) graph, and clusters were called with the Louvain algorithm (resolution = 0.5). Uniform manifold approximation and projection (UMAP) based on the same PCs provided two-dimensional visualization. Marker genes were defined using min.pct = 0.25 and |log_2_FC| > 0.25.

Natural-killer (NK) cells were identified with SingleR in combination with celldex::DatabaseImmuneCellExpressionData () [13], yielding 548 – 939 NK cells per sample after filtering. To resolve NK-cell heterogeneity, we extracted the normalized NK-cell expression matrices, transposed them (rows = cells, columns = genes), and performed unsupervised K-means clustering with scikit-learn (v 1.4.0) [14]. Candidate cluster numbers k = 2 – 10 were evaluated, and the solution with the highest average silhouette score was chosen; clustering was run with random_state = 0 for full reproducibility. Outliers were defined as cells lying more than 2 SD beyond the mean Euclidean distance to their cluster centroid. Cluster structure was visualized with t-distributed stochastic neighbor embedding (t-SNE) [15], and plots were generated in matplotlib [16]. Cluster assignments and outlier flags are provided as Supplementary Data.

Gene-set enrichment analysis (GSEA v 4.3.2) [17] was carried out against the Hallmark collection [18] of the Molecular Signatures Database (MSigDB) [19]. Differential-expression testing between NK-cell clusters (excluding genes used for K-means clustering) was performed on log-transformed counts with a two-sample, two-tailed Student’s t-test. We used the CellChat R package (version 1.6.1) [17] to infer intercellular communication networks from our normalized NK cell expression data.

## Results

The three cases have been followed at CHOP since birth. The first case is a 13-year-old male with HLHS who has undergone multiple staged cardiac surgeries, including the Fontan and Sano procedures. His clinical course is complicated by feeding difficulties necessitating gastrostomy tube (G-tube) feeding, vocal cord paralysis, developmental delays, and recurrent respiratory infections. He has also experienced atrial flutter, thrombosis, and multiple allergies, requiring ongoing medical management. The second case is a 15-year-old male with HLHS and additional congenital heart defects, including atrioventricular canal and coarctation of the aorta. He has undergone the Fontan and hemi-Fontan operations and presents with feeding difficulties, gastroesophageal reflux, and a hypercoagulable state. Frequent embolic events necessitate chronic anticoagulation therapy. His family history includes congenital anomalies and asthma. The third case, a female who died at age 11, had a single ventricle physiology with an unbalanced atrioventricular canal, Tetralogy of Fallot, congenital heart block, and heterotaxy syndrome. She underwent a bidirectional Glenn shunt and pacemaker placement. Her clinical course was further complicated by developmental delay, feeding difficulties, chronic lung disease, pulmonary hypertension, and recurrent wound complications, requiring nasogastric tube feeding and long-term cardiovascular support.

### Heterogeneous NK cell clusters with amplified changes of transcriptomic profiles in SV/HLHS

**FIGURE 1:**
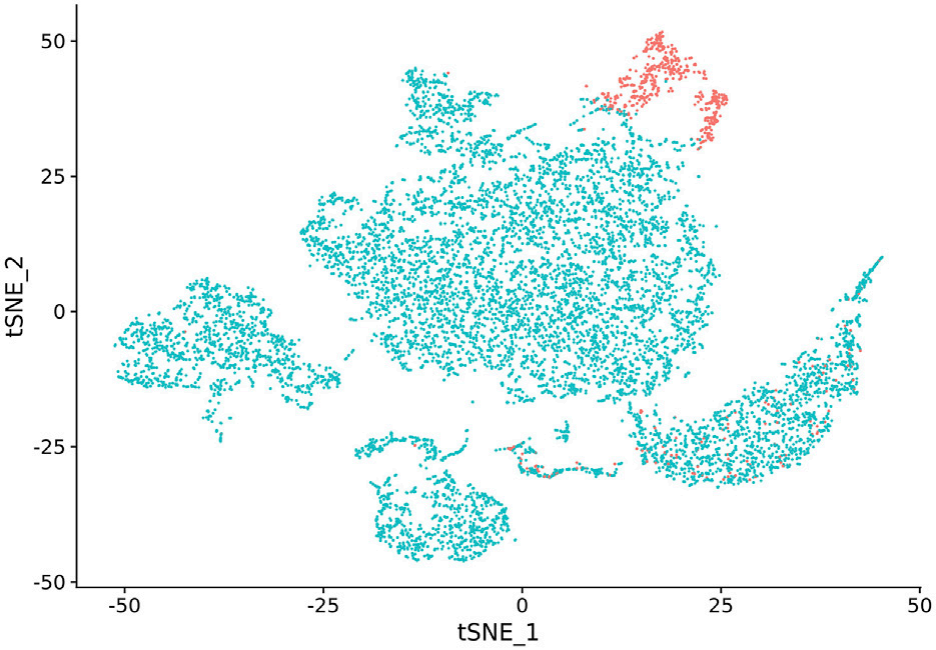
NK cell cluster versus other cell types visualized by t-SNE. NK cells are highlighted in red.

**FIGURE 2:**
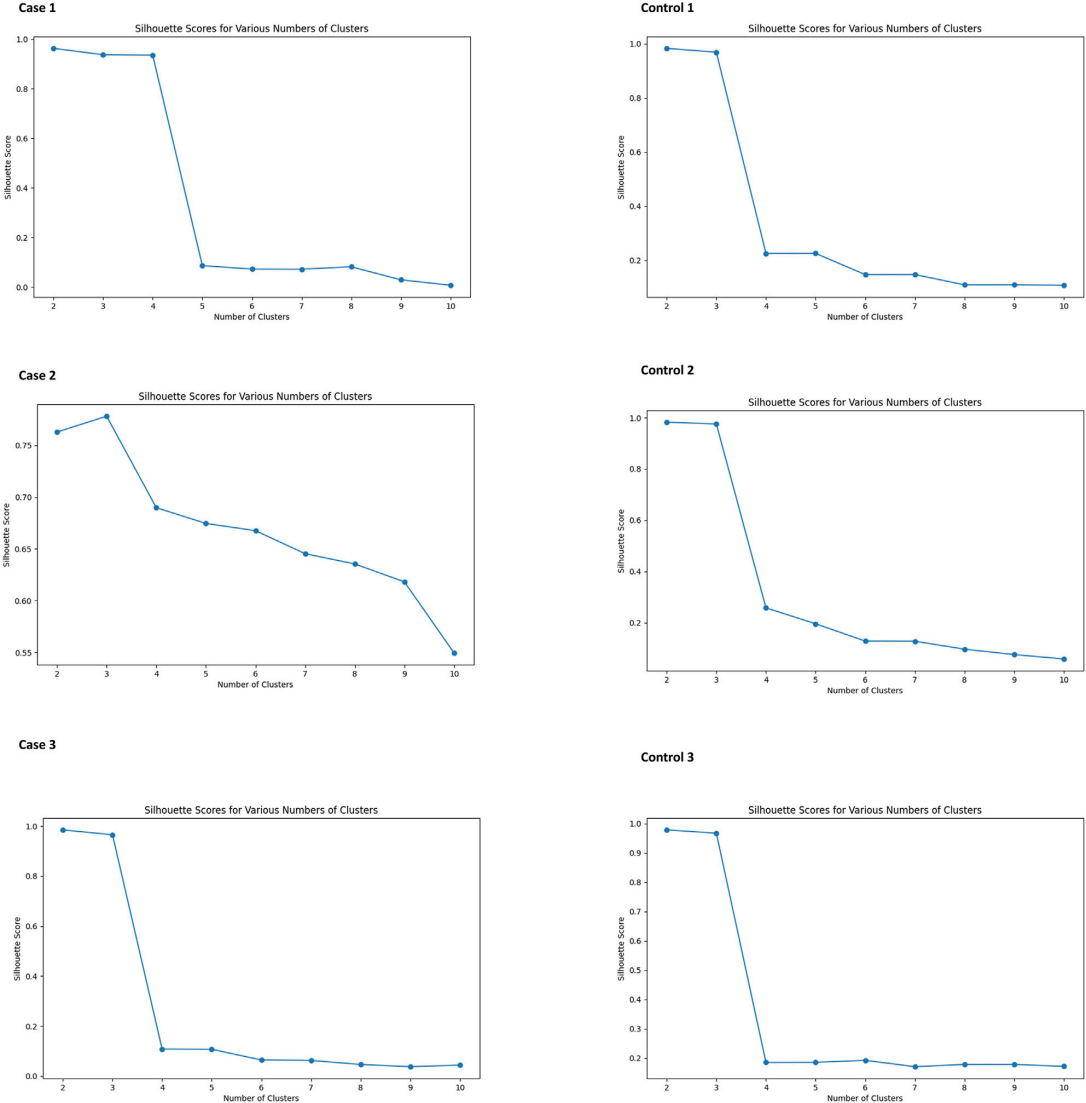
Silhouette scores for various numbers of clusters in K-means analysis of NK cells. All subjects show high Silhouette scores with two clusters.

**FIGURE 3:**
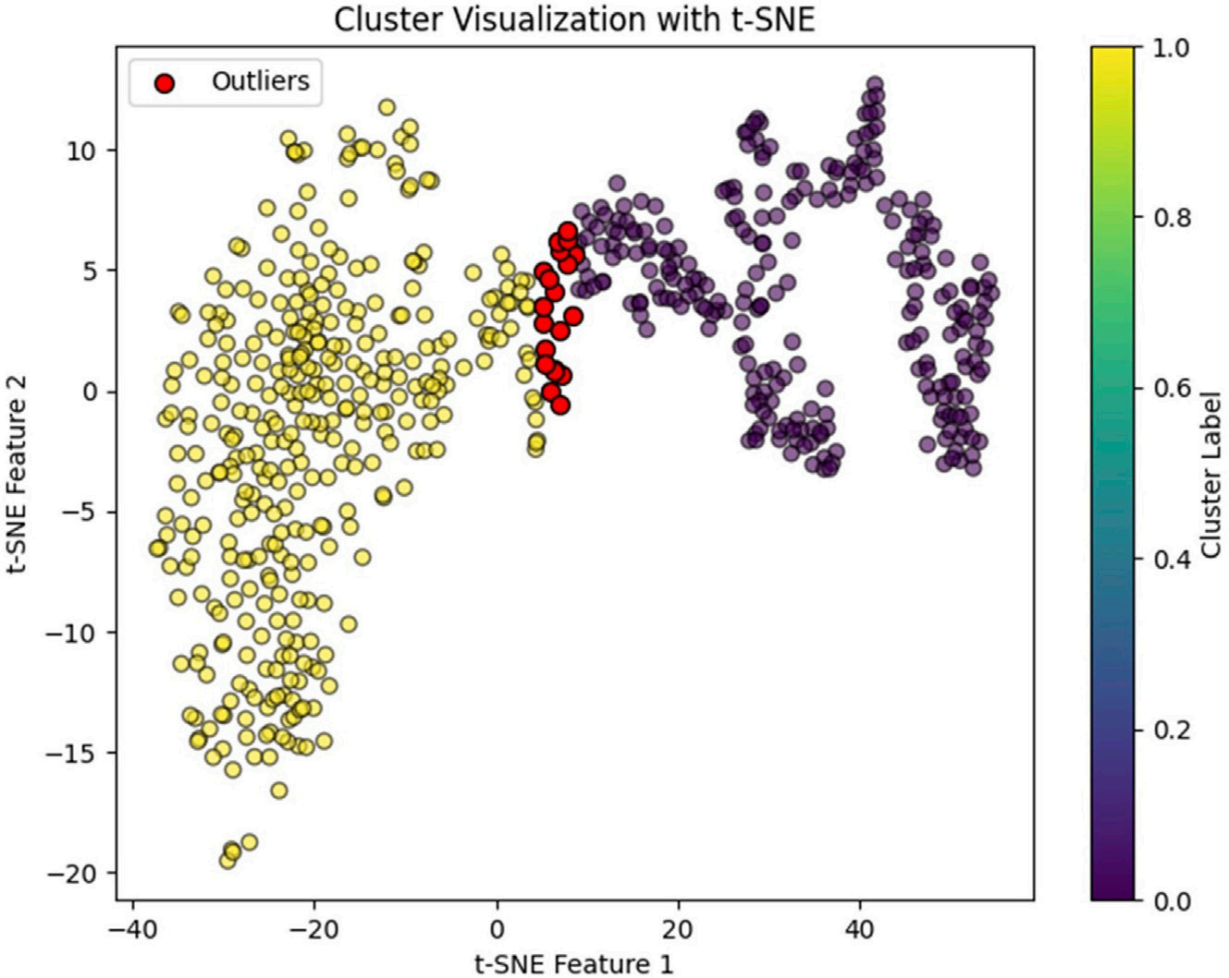
NK cell clusters visualized by t-SNE. Red dots indicate identified outliers.

In our study, we observed that NK cell clusters tend to be heterogeneous (Figure 1). With their function still unknown, there are no definitive gene markers available to classify these NK subpopulations with conventional scRNA-seq pipelines or traditional flow cytometry. To test whether these heterogeneous NK cells are responsible for the DE genes, we examined the 1600 DE genes identified in NK cells from our previous study [9]. K-means clustering with these DE genes yielded high Silhouette scores with 2 clusters in all the 6 samples (Figure 2). As shown in Figure 3, the NK cells within the same sample can be divided into two distinct clusters. Despite clinical heterogeneity among the cases, including differences in anatomical diagnoses, surgical histories, and comorbid conditions, we observed consistent NK cell clustering patterns and transcriptomic signatures across all three samples, suggesting a potentially conserved NK cell transcriptomic signature in SV/HLHS. We then performed GSEA using the averaged difference between the two clusters across the three cases (Table 1; Supplementary Table S1). In one cluster, the gene sets HALLMARK_ANDROGEN_RESPONSE and HALLMARK_HYPOXIA are significantly upregulated, and HALLMARK_HEME_METABOLISM is significantly downregulated.

**TABLE 1:** Gene sets with statistical significance identified by GSEA analysis.

HALLMARK gene set	Size	ES	NES	NOM p-val	FDR q-val
1,600 genes for cell clustering with differential expression in NK cells associated with SV/HLHS					
Cluster 1 vs. cluster 0: upregulated					
ANDROGEN_RESPONSE	16	0.81	1.97	0	0
HYPOXIA	20	0.66	1.64	0.009	0.041
Cluster 1 vs. Cluster 0: downregulated					
HEME_METABOLISM	23	−0.74	−1.74	0	0.004
Cluster expression in Cases vs. Controls: upregulated					
ANDROGEN_RESPONSE	16	0.81	1.94	0	0.004
OXIDATIVE_PHOSPHORYLATION	52	0.63	1.8	0.001	0.021
INTERFERON_ALPHA_RESPONSE	23	0.68	1.73	0.006	0.031
INTERFERON_GAMMA_RESPONSE	38	0.63	1.71	0.004	0.027
Cluster expression in Cases vs. Controls: downregulated					
HEME_METABOLISM	23	−0.65	−1.9	0.005	0.03
789 genes with differential expression between two clusters in NK cells^a^					
Cluster 1 vs. Cluster 0: upregulated					
ALLOGRAFT_REJECTION	25	0.68	1.54	0	0.022
Cluster 1 vs. Cluster 0: downregulated					
UV_RESPONSE_DN	15	−0.43	−1.58	0	0
Cluster expression in Cases vs. Controls: upregulated					
Nonsignficant					
Cluster expression in Cases vs. Controls: downregulated					
UV_RESPONSE_DN	15	−0.85	−3.06	0	0

^a^ These genes have not been identified as differentially expressed in NK, cells associated with SV/HLHS, and were not used for cell clustering.

ES: enrichment score; NES: normalized enrichment score; NOM p-val: nominal p-value; FDR: false discovery rate.

Compared to the controls, the upregulated androgen response and downregulated heme metabolism are more significant in the cases, as shown by Cluster expression in Cases vs. Controls in Table 1. In addition, upregulation of the gene sets HALLMARK_OXIDATIVE_PHOSPHORYLATION, HALLMARK_INTERFERON_ALPHA_RESPONSE, and HALLMARK_INTERFERON_GAMMA_RESPONSE are more significant in Cluster 1.

### Other gene expression in the two clusters of NK cells

To further characterize the two NK cell clusters, we examined the expression of the two NK cell clusters for genes not used for the clustering. We identified 789 genes with DE between the two clusters in all three cases (Supplementary Table S2). GSEA analysis showed upregulated HALLMARK_ALLOGRAFT_REJECTION and downregulated HALLMARK_UV_RESPONSE_DN in Cluster 1 in cases. The downregulated HALLMARK_UV_RESPONSE_DN in Cluster 1 in cases is more significant than that in controls (Table 1). CellChat-inferred ligand–receptor interactions between Cluster 0 and Cluster 1 are listed in Supplementary Table S3.

## Discussion

In this study, we identified heterogeneous NK cell clusters with amplified transcriptomic changes in SV/HLHS. These gene-expression differences may not only arise as consequences of the cardiac condition but may also shape distinct clinical trajectories, offering potential transcriptomic features and intervention points for infection control, thrombosis, and protein-losing enteropathy.

### Distinct functional NK cell populations

In our analysis of NK cell populations, Cluster 1 exhibits distinct transcriptional profiles compared to Cluster 0 in term of the DE genes associated with SV/HLHS, marked by significant upregulation of gene sets including HALLMARK_ANDROGEN_RESPONSE and HALLMARK_HYPOXIA, alongside a notable downregulation of HALLMARK_HEME_METABOLISM. Androgen signaling may modulate NK cell immune functions, potentially tempering their cytotoxic activity while promoting survival and adaptation, particularly under stress [20]. Concurrently, the increased expression of hypoxia-responsive genes likely enhances NK cells' capability to operate in oxygen-deprived environments [21]. The reduced heme metabolism complements the other observed changes by potentially reducing oxidative stress and regulating metabolic responses, including oxidative phosphorylation, amino acid, and xenobiotic metabolism [22], likely shifting towards more energy-conserving processes and potentially linking Cluster 1 to the elevated thrombotic risk observed clinically. Collectively, these transcriptional changes suggest that NK cells in Cluster 1 are likely geared towards adaptation and survival in hypoxic or stress-related conditions. In contrast, Cluster 0 with different gene expression patterns might retain a higher cytotoxic capability and responsiveness, better suited for environments requiring rapid and robust immune reactions without the adaptive pressures of chronic stress.

The distinct overexpression of cytotoxic effector genes *NKG7*, *GNLY*, *GZMB*, and *FGFBP2* in Cluster 1 versus key transcription factors *ZEB1*, *BACH2*, and *BCL11B* in the other reflects divergent, subtype-specific regulatory networks and stable epigenetic landscapes. Studies show *BACH2* loss drives a discrete cytotoxic program with increased granzymes rather than a gradual shift [23], *ZEB1* expression is associated with maintenance/less-cytotoxic states by repressing effector loci [24], and *BCL11B* drives a distinct adaptive-like differentiation incompatible with the cytotoxic program [25]. In addition, epigenetic analyses reveal fixed chromatin states for each subtype [26], and single-cell clustering consistently uncovers separate NK endpoints with no intermediate transcriptional profiles [27]. These marker genes show differential expression in opposite directions between clusters, emphasizing their divergent functional programs without evidence of a gradual transition pattern. Together, these patterns support the interpretation that the subtypes represent two functionally opposite, terminal NK cell states rather than stages along a developmental or activation trajectory.

In addition, our cell–cell communication analysis revealed that the cytotoxic cluster (Cluster 1) preferentially engages inhibitory checkpoints such as HLA-E binding to the CD94/NKG2A heterodimer [28, 29] and TGFB1 signaling through TGFβR1/2 [30], suggesting built-in mechanisms to restrain potent effector activity, whereas the transcription factor–high cluster (Cluster 0) shows alternative contact-mediated interactions (e.g., CLEC2–KLRB1) [31], consistent with niche or maintenance signals. These divergent communication patterns align with the distinct overexpression of the DE genes described above. Together, they reinforce that each subtype represents a terminal NK cell state with its own regulatory network and signaling milieu.

### Distinct adaptations in cluster 1 NK cells in SV/HLHS patients

The two clusters of NK cells observed in our study, present in both healthy controls and cases, likely represent distinct functional states tailored to specific immunological needs. Notably, marked differences in the expression profiles of these clusters between cases and controls were observed, as shown by Cluster expression in Cases vs. Controls in Table 1. Cluster 1 in the cases shows significantly heightened androgen response and more pronounced downregulation of heme metabolism compared to controls. These changes may serve a dual role. This modulation could be protective, reducing potential tissue damage in chronic disease contexts such as seen in SV/HLHS. On the other hand, this reduced cytotoxicity might compromise the NK cells' ability to clear pathogens effectively, potentially increasing the risk of infections. In addition, upregulation of the gene sets related to oxidative phosphorylation, and interferon responses, both alpha and gamma, are more significant in Cluster 1 in cases. Enhanced oxidative phosphorylation indicates that these NK cells have elevated energy production capabilities [32]. Upregulated Interferon responses imply this cluster of NK cells is primed for responding to viral infections and potentially other pathogens [33]. Altogether, upregulated HALLMARK_ANDROGEN_RESPONSE and downregulated HALLMARK_HEME_METABOLISM, plus these additional adaptations, suggest strategic modifications of NK cells to meet the demands of specific pathological states in prolonged exposure to pathogens or inflammatory conditions in SV/HLHS. These findings raise the possibility that pharmacologic modulation of these pathways could help restore a more balanced NK-cell phenotype, enhancing cytotoxicity while preserving protective adaptation in SV/HLHS.

### Further characterization of the two NK cell clusters

In our extended analysis, we examined additional gene expressions within the two NK cell clusters beyond those initially used for clustering. This examination revealed 789 genes with DE between the two clusters across all cases. Specifically, Cluster 1 in cases exhibited upregulated HALLMARK_ALLOGRAFT_REJECTION and downregulated HALLMARK_UV_RESPONSE_DN. The upregulation of the allograft rejection gene set in Cluster 1 underscores a heightened immunological readiness to recognize and attack non-self cells, a crucial feature for defending against foreign tissues and potentially harmful pathogens [34]. The downregulation of genes associated with the UV response, including key regulators like *DYRK1A, ATXN1, ATP2B1, NIPBL, RUNX1*, and *SIPA1L1*, presents a complex scenario. These genes are crucial for various NK cell functions such as signaling, development, and effector responses [35, 36]. Their reduced expression might impair the NK cells’ capacity for regular immune surveillance and cellular migration. Furthermore, the downregulation of genes associated with the UV response in cases is significantly higher than controls. When considered together, these opposing trends in gene expression paint a picture of a dual nature in NK cells in Cluster 1, which could render these NK cells less effective in handling routine immune tasks, posing risks for viral infections and complicate the management of SV/HLHS.

A focused transcriptomic signature derived from these findings could be developed for longitudinal monitoring, with potential to correlate expression dynamics with postoperative infection rates and lymphatic complications. Functional assays will also be critical to determine whether restoring regulators like *RUNX1* can reverse migratory defects without exacerbating inflammation. This complexity may underlie both the heightened inflammatory tone and reduced immune resilience seen in SV/HLHS patients.

## Conclusion

Overall, these findings provide an initial gene expression profile of NK cells in SV/HLHS, suggesting potential adaptations that may support survival in a challenging physiological environment. However, these adaptations could also compromise the cells' ability to manage additional stresses, such as infections and inflammation. Given that this analysis is based solely on gene expression data without proteomic or functional validation, the conclusions should be interpreted with caution. Further studies, incorporating proteomics and functional assays, are necessary to fully understand the immune implications and to develop targeted strategies that support immune function without exacerbating the underlying condition. A longitudinal profiling of NK cells over time, rather than a single timepoint, would provide valuable insights into their role in disease progression and adaptation.

One limitation of this study is the small sample size. However, the consistent identification of two distinct NK cell clusters in all three healthy controls, along with statistically significant findings, supports the robustness of our results. Another limitation is the current lack of knowledge regarding the subpopulations of NK cells in peripheral blood. We hope this study brings attention to the heterogeneity of NK cells in peripheral blood and encourages further research into these subpopulations. An additional important limitation is the clinical heterogeneity among the cases, including differences in sex, associated cardiac anomalies, surgical interventions, and comorbidities such as developmental delays and hypercoagulable states. This heterogeneity reflects the complexity of SV/HLHS patients and poses a challenge to isolating disease-specific transcriptomic signatures. Nonetheless, the reproducibility of NK cell clustering and the consistent enrichment of specific gene expression pathways across all cases support the presence of a shared transcriptomic program. Future studies with larger, stratified cohorts will be essential to investigate how specific clinical variables may shape immune cell behavior.

## Data Availability

All data generated or analyzed during this study are included in the article and its Supplementary Material. The raw sequencing datasets presented in this article are not readily available because of privacy and ethical restrictions; requests to access these datasets should be directed to the corresponding author.
